# Molecular allergology

**DOI:** 10.1186/1824-7288-40-S1-A78

**Published:** 2014-08-11

**Authors:** Roberto Bernardini, Vincenzo Ragazzo, Cinzia Cioni, Rosa Cervone

**Affiliations:** 1 Pediatric Unit, San Giuseppe Hospital, Empoli, Italy

## Background

Allergy diagnosis is based on the history, clinical symptoms and physical examination of the patient in combination with results of in vivo and in vitro test. Today the allergy diagnosis can be refined using molecular allergology, which allows the quantification of allergen specific IgE antibodies to a single, pure allergen molecule (component). This contributes to a more exact diagnosis, and thereby enables a more accurate disease prognosis in terms of tolerance development or risk assessment leading to an improved patient management. Depending on how unique the components are in distribution and structure, they are classified as Specific or Cross-reactive. Specific allergen components are more or less unique for their source, and are found only in a rather limited number of very closely related species. In each allergen sources there are one or more specific allergen components and sensitization to these indicate a true/genuine sensitization, meaning that the corresponding allergen source is the primary cause of the clinical symptoms. Cross reactive allergens on the other hand, are widely distributed (also in distantly related species) and due to their high degree of structural similarity may cause IgE antibody cross-reactivity. Components can be either labile or stable depending on their structure. Stable proteins are not easily broken down by cooking, processing or by enzymes in the saliva and in the gut. Stable proteins will reach the circulation in a more or less intact form and therefore potentially give rise to systemic reactions. Labile proteins that are easily broken down by processing, cooking or by enzymes in the saliva or gut will mainly give rise to local reactions, such as Oral Allergy Syndrome.

## Conclusions

The Molecular Allergology allows: 1) To identify the right patients for Specific Immunotherapy: sensitization to specific allergen components is essential for successful Specific Immunotherapy. By matching patients having a genuine sensitization with an extract from the relevant source, treatment outcome is improved. 2) To explain symptoms due to cross-reactivity: symptoms elicited by cross-reacting antibodies can be distinguished from those caused by genuine sensitization which is important for patient management and for giving adequate avoidance advice. In cases where only cross-reactive sensitization is identified, further testing to find the primary sensitizer should be undertaken. 3) To assess the clinical risk for reaction: sensitization to allergen components that are stable may elicit systemic reactions, as well as local reactions, while sensitization to labile components is connected mainly with local reactions.

